# Canned complementary porridges for infants and young children (6–23 months) based on African indigenous crops; nutritional content, consistency, sensory, and affordability compared to traditional porridges based on maize and finger millet

**DOI:** 10.1111/mcn.13752

**Published:** 2024-11-05

**Authors:** Trond Løvdal, Josefine Skaret, Gorana Drobac, Blessed Okole, Izumi Sone, Natalia Rosa‐Sibakov, Paula Varela

**Affiliations:** ^1^ Department of Process Technology Nofima—Norwegian Institute of Food, Fisheries and Aquaculture Research Stavanger Norway; ^2^ Department of Consumer & Sensory Science Nofima—Norwegian Institute of Food, Fisheries and Aquaculture Research Ås Norway; ^3^ Advanced Agriculture and Food Cluster Council for Scientific and Industrial Research (CSIR) Pretoria South Africa; ^4^ VTT Technical Research Centre of Finland Espoo Finland

**Keywords:** African indigenous crops, child nutrition, complementary foods, food and nutrient insecurity, infant and young children feeding, protein/energy nutrition

## Abstract

Child malnutrition is a major health problem in Sub‐Saharan Africa. Complementary foods made from African indigenous and locally available raw materials are often low in protein and nutrients. It is, therefore, important to supply complementary foods that are nutritious and affordable, and with an acceptable consistency and taste. The objective of this study was to develop, on a pilot scale, food‐to‐food fortified, convenient, canned complementary porridges based on blends of African indigenous crops, i.e., orange fleshed sweet potato (OFSP) flour, and leguminous (i.e., cowpea, and Bambara groundnut) and cereal flours (i.e., teff, finger millet, maize, and amaranth), and milk powder. Plant‐based, African complementary foods are often lacking in vitamin A, zinc, iron, and energy. Porridge with OFSP on a 32% dry weight (dw) basis achieved recommended levels of vitamin A (530 µg per 100 g dw). Satisfactory energy (431 Kcal per 100 g dw) was obtained by supplementation of vegetable oil. A nutritious, low‐cost porridge (costing 0.15 € per 100 g can) that fulfills consistency constraints was obtained by including supplements of zinc and iron salts as ingredients. The solids content and thus protein/energy could be significantly increased using protein fractionated or germinated cowpea flours without compromising on viscosity. The sensory profile was characterised by more intense vegetable, leguminous, and malty flavours as compared to traditional reference porridges.

## Key messages

- In developing countries, complementary foods are often plant‐based, which may lead to deficiencies of e.g., vitamin A, zinc, and iron, and does not support sufficient energy. This study shows that vitamin A content can be increased by use of Orange Fleshed Sweet Potato (OFSP) flour as an ingredient in complementary porridges. Mineral‐rich foods are expensive, and with only local crops available, a minimum cost is hardly achieved without supplementation with cost‐limiting nutrients zinc and iron salts.
- Fat content may be improved by whole milk powder and vegetable oils, also leading to more energy. Solids content, and hence energy and protein, may be increased by the use of protein fractionated and germinated legume flour, ‐ without compromising on viscosity.
- The present study shows that low‐cost complementary nutritious porridges that fulfills the consistency constraints can be developed based on indigenous African crops by including supplements of zinc and iron salts.

## Graphical abstract

Affordable and nutritious canned complementary porridges, based on indigenous African crops, that are high in e.g., zinc, iron, and vitamin A, can be industrially manufactured. The porridges fall within the recommended range regarding consistency and their taste is comparable to traditional porridges.

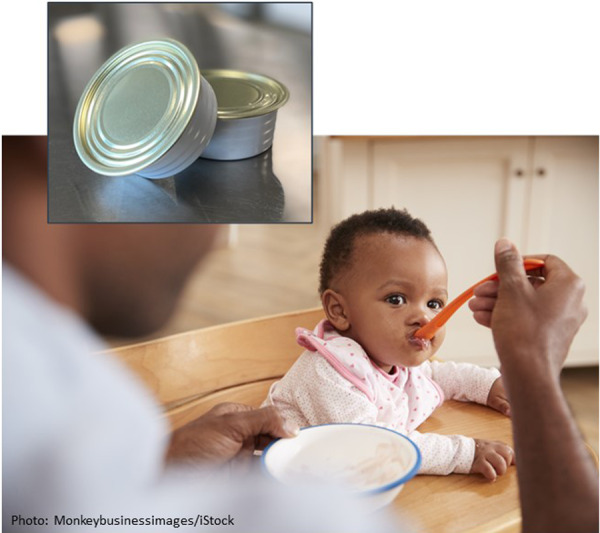

## 1 INTRODUCTION

Malnutrition includes over‐ and under‐nutrition, and the deficiency of micronutrients essential for normal growth and development, which leads to the so‐called triple burden of malnutrition. Child malnutrition is a leading risk factor for human health, and undernutrition and micronutrient deficiencies are emerging in most sub‐Saharan African regions, in parts of Asia and the Caribbean, and also in other parts of the world (Murray et al., 2020; Nel, 2022). Child malnutrition is mainly due to inadequate diet during pregnancy and the first 2 years of life (also known as the first 1000 days in the lifecycle [Black & Heidkamp, 2018]). Maternal undernutrition generally results in foetal underweight at birth. Moreover, inadequate breast‐feeding as well as inappropriate and non‐affordable formula milk and complementary food are major factors that contribute to malnutrition in children (Issaka et al., 2017). Complementary foods made from indigenous and locally available cereal grains and root crops are often low in protein and of low protein quality (Kikafunda et al., 1997; Temba et al., 2017; Thaoge et al., 2003). Viscous porridge made from maize, cassava, or sorghum has to be diluted to enable small children to swallow, and the dilution causes insufficient energy and protein (Makame et al., 2019; Muoki et al., 2012; Sayre et al., 2011). It is, therefore, important to supply food items for this age group that are both nutritious and affordable, and with an adequate viscosity. Such foods need to be rich in protein and energy and support enough essential micronutrients and vitamins. In many countries, the period of complementary feeding from 6 to 23 months is the time of peak incidence of growth faltering and micronutrients deficiencies (Dewey & Adu‐Afarwuah, 2008). Iron deficiency is reported as a problem in this age group, as well as calcium, zinc, folate, vitamin A, B1, D, B12, etc. (Black, 2014; Murray et al., 2020). Proper nutrition during infancy and towards the age of two is critical to ensure optimal physical and mental development of infants and young children. The World Health Organization (WHO) Global Strategy on Infant and Young Child Feeding (World Healt Organization, 2002) noted that industrially processed foods are an option for parents who can afford them and have the knowledge and facilities to safely prepare and feed them.

African crops, such as orange fleshed sweet potato (OFSP), cowpea, Bambara groundnut, teff, maize, finger millet, and amaranth vary in nutritional composition. Cowpea and Bambara groundnut are oil‐rich and rich in protein (Goncalves et al., 2016; Jayathilake et al., 2018; Tan et al., 2020; Temba et al., 2017), OFSP is rich in β‐carotene and provitamin A (Amagloh & Coad, 2014; Neela & Fanta, 2019), whereas the cereal crops are rich in e.g., iron, zinc, and vitamins (Abebe et al., 2007; Gebre, 2019). Thus, it may be beneficial to combine them in complementary infant meals to achieve nutritious diets from a food‐to‐food fortification perspective. The children's ability to orally process thick and sticky porridges, which is typically a feature of most common African indigenous porridges based on cassava, maize and sorghum, is limited (Makame et al., 2019). To achieve a protein dense and energy‐rich meal with an acceptable viscosity, OFSP or other low‐viscosity flours can be added to the porridge recipe (Makame et al., 2020). According to De Carvalho et al. (2015), it may be argued that food consistency is the most important technological property of foods for children at risk of malnutrition or starvation.

With the backdrop that iron, zinc, and vitamin A deficiencies are estimated to affect 16%, 39%, and 42%, respectively, of children <5 years in Africa (Black, 2014), and that foods supplying sufficient zinc is ubiquitously unaffordable and only a few foods are affordable sources of iron, whereas the poorest households cannot afford vitamin A‐rich foods (Ryckman et al., 2021), approaches to reduce prices of unaffordable nutrients are needed.

The goal of the study was to develop nutritious, convenient, and affordable complementary porridge for infants based on indigenous and locally available African crops considering the constraints posed by consistency and sensory properties.

## 2 MATERIALS AND METHODS

### 2.1 Flours raw material

Flour of OFSP, cowpea, and Bambara groundnut were imported from local producers in South Africa, while maize (cv Longe 8), finger millet (cv Naromill), and amaranth grain flours were imported from Uganda. Teff flour was of the light type and was procured by online shopping (Da Carla, Sweden). All recipes were prepared with skimmed milk powder (TINE, Norway) and tap water. The skimmed milk powder was made from low‐pasteurised skimmed cow milk by spray drying and had a whey protein index between 1.5 and 6 mg undenatured whey protein per gram of powder. The energy content of skimmed milk powder was 345 Kcal per 100 g, and the fat, protein, and carbohydrate content were 0.8, 36.9, and 47.9 g/100 g, respectively. Unmodified flour raw materials are presented in Appendix S1, Table S1.

### 2.2 Porridge recipes and preparation

All canned complementary porridges were prepared as follows: 700 g of water was cooked to boiling. In a separate container, 200 g of cold water was mixed with the dry ingredients. The two liquids were mixed with continuous stirring, brought to boil, and kept at careful boiling for 3 min. The porridges were set aside and allowed to soak for 5 min. The porridges were then transferred to small cans (100 g volume). The cans were sealed and sterilised in an autoclave and the cans became shelf stable and could be stored at room temperature. The autoclave programme resulting in a F_0_‐value of 22 is shown in Table 1. The reference porridges were prepared the same way, except that they were boiled for 7 min instead of three, and not autoclaved. References were prepared fresh before analysis. Solids content in the porridges was between 12.2% and 12.6%.

**Table 1:** Autoclave programme for infant porridges (Tropical programme).

Phase	Temperature,°C	Pressure, bar	Time, min
Heating	90	0.5	2
Heating	116	1.7	5
Heating	116	1.7	60
Cooling	60	1.5	5
Cooling	30	0.4	5
Cooling	20	0.2	10

### 2.3 Part 1: Prototypes profiling

After an initial screening of several recipes, six recipes for canned complementary porridges were selected for further analysis based on rheology properties with viscosity as the primary constraint, and calculated protein and energy content as the secondary constraint, and informal sensory (taste) tests as the third constraint. Two references, made from maize and African indigenous finger millet flour, respectively, with similar solids content and viscosity as the six experimental porridges were prepared by traditional boiling. The recipes for the porridges are shown in Table 2.

**Table 2:** Ingredients in recipes for canned infant porridges and references.

Ingredient	Porridge no.							
	#1	#2	#3	#4	#5	#6	Ref 1	Ref 2
OFSP (g)	40	20	10	10	5	5	–	–
CP (g)	25	10	–	–	–	–	–	–
BGN (g)	–	–	15	15	–	–	–	–
Teff (g)	40	40	40	–	–	15	–	–
Maize (g)	–	20	25	25	60	30	–	90
FM (g)	–	–	–	40	–	15	90	–
Amaranth (g)	–	–	–	–	25	25	–	–
SMP (g)	20	40	40	40	40	40	40	40
Water (g)	900	900	900	900	900	900	900	900

Abbreviations: BGN, Bambara groundnut flour; CP, cowpea flour; FM, finger millet flour, OFSP, orange fleshed sweet potato flour; SMP, skimmed milk powder.

### 2.4 Part 2: Optimisation, enrichment and supplementation

Based on the results from Part 1: prototypes profiling, prototype #1 was used as basis for the new set of porridges. Prototype #1 was superior in β‐carotene, and hence provitamin A, which is one of the cost‐limiting nutrients, and otherwise comparable to the others in energy, macro‐nutrients, and with a beneficial consistency (see Section 3). Increasing the solids content of the porridges will generally lead to increased protein and energy content, and this was achieved without compromising the consistency using the protein fraction of cowpea flour (PF‐CP), and the use of germinated cowpea flour (G‐CP).

PF‐CP and G‐CP flour were prepared as previously described by Palsola (2022). Briefly, PF‐CP was prepared with a 50‐ATP classifier (Hosokawa Alpine AG) with a rotor speed of 9000 rpm and 50 m^3^ h^−1^ airflow. The germination process was initiated by soaking whole cowpeas in water at 25°C for 2 h, dried for 30 min, and then soaked again at the same temperature for 8 h. After that, the cowpea was allowed to germinate for 32 h at 25°C, followed by drying for a total of 21 h with stepwise temperature increase from 50°C to 83°C. After drying, the sprouts were removed mechanically, and the cowpea was milled twice by pin disc milling (17,800 rpm) with Hosokawa Alpine 100UPZ‐lb fine impact mill (Hosokawa Alpine AG). The macro nutrients of PF‐CP and G‐CP is shown in Appendix S1, Table S2.

Porridges with two levels of PF‐CP (#1—PF‐CP 20% and #1—PF‐CP 33%) and one level of G‐CP (#1—G‐CP 50%), were prepared. The levels of PF‐CP and G‐CP inclusion were based on rheology analysis. In #1—PF‐CP 20%, unmodified cowpea was substituted 1:1 with PF‐CP to result in a PF‐CP content of 20% on a dry weight (dw) basis. In #1—PF‐CP 33%, PF‐CP substituted unmodified cowpea and was increased to 33% of the dw, and all other ingredients were kept constant, resulting in an increase in solids content from 12.2% to 14.3%. In #1—G‐CP 50%, unmodified cowpea was not used, and G‐CP was added to 50% of the dw, leading to an increase in solids content from 12.2% to 18.2%.

To better achieve recommended nutrient levels for complementary foods as proposed by Lutter and Dewey (2003) and CODEX CAC/GL 08 (1991), prototype #1 was used as a basis for an improved prototype called #1—Enriched, with skimmed milk powder substituted with whole milk powder (Tine) with an energy content of 545 Kcal per 100 g, and fat, protein, and carbohydrates content 35.0, 24.1, and 31.1 g/100 g, respectively, and fortified with sunflower oil (Eldorado, Unil) with an energy content of 899 Kcal per 100 g, and fat content of 99.9 g per 100 g. Whole milk powder and sunflower oil were both added to increase fat and thus energy content. Additionally, #1—Enriched was supplemented with zinc citrate and iron sulphate according to De Carvalho et al. (2015). The composition of porridges with modified flour and the enriched porridge are shown in Table 3. These porridges were prepared and processed as explained above for the initial six prototypes.

**Table 3:** Ingredients in recipes for protein‐optimised and enriched canned complementary porridges.

Ingredient	Porridge no.			
	#1—PF‐CP 20%	#1—PF‐CP 33%	#1—G‐CP 50%	#1—Enriched
OFSP (g)	40	40	40	40
CP (g)	–	–	–	25
PF‐CP (g)	25	50	–	–
G‐CP (g)	–	–	100	–
Teff (g)	40	40	40	40
SMP (g)	20	20	20	–
WMP (g)	–	–	–	20
Sunflower oil (g)	–	–	–	7.5
Zinc citrate (mg)	–	–	–	130
Iron sulphate (mg)	–	–	–	65
Water (g)	900	900	900	900

Abbreviations: CP, cowpea; G‐CP, germinated CP; OFSP, orange fleshed sweet potato; PF‐CP, protein‐fraction of CP; SMP, skimmed milk powder; WMP, whole milk powder.

### 2.5 Nutrient analysis

Porridge samples (100 g) were freeze‐dried with a Scanvac CoolSafe Touch Superior (LaboGene) with an ice condenser temperature of −50°C and pressure of 0.37 hPa and stored at −20°C in the dark until analysis. Crude protein content was analysed by the Kjeldahl method according to ISO 5983‐2. Fat (Soxleth) was analysed according to AOCS Ba 3‐38. Crude fibre was analysed according to AOCS Ba 6a‐05. Phosphorus, iron, potassium, calcium, sodium, and zinc were analysed according to the European Standard EN 15621 (European Committee for Standardization, 2017). Available carbohydrate was calculated by subtraction (100 ÷ crude protein ÷ crude fibre ÷ crude fat), and the energy value of the porridges was calculated as described by FAO/WHO (1998) Analytical Methods for Carbohydrates in Foods as [(4 × carbohydrate) + (4 × protein) + (9 × fat)] and energy value expressed in Kcal/100 g. Two (Part 1) or three (Part 2) parallel cans were analysed for the analyses mentioned above.

Extraction of ascorbic acid was performed by addition of 5 mL 4.5% *m*‐phosphoric acid to 0.5 g of sample material followed by homogenisation by use of a vortex mixer and incubation in an ultrasound bath for 5 min. After extraction, the samples were centrifuged, and 1 mL of the extract was passed through a 1 mL (50 mg) solid phase extraction (SPE) cartridge (SilactSPE C18, Affinisep, Teknolab AS). The non‐retarded eluate was analysed by use of high‐performance liquid chromatography (HPLC; Agilent 1100‐system with diode‐array detector, Agilent Technologies, Matriks AS) over a Supelcosil LC‐18 column, 25 cm × 4.6 mm, 5 µm (Supelco) according to Sánchez‐Mata et al. (2000). The analysis was calibrated against authentic ascorbic acid (95212, Fluka, Sigma Aldrich).

β‐carotene was extracted from 0.5 g sample material by using a saponification process followed by liquid‐liquid partition against methyl‐*tert*‐butyl ether (MTBE). In short, the samples were mixed with 2 mL of a 50% NaOH solution and kept at 70°C for 1 h protected against light. The samples were then centrifuged, and the liquid phase was then partitioned against 3 × 2 mL MTBE. Pooled extracts (6 mL) were dried under N_2_ gas and dissolved in 0.5 mL methanol. The samples were filtered, and β‐carotene content was analysed by using HPLC supplied with an ODS Hypersil column (10 cm × 3 mm, 3 µm, Supelco) according to Gupta et al. (2015). The analysis was calibrated against authentic β‐carotene (C‐9750, Sigma Aldrich). Vitamin A was expressed as retinol equivalents (RE) and estimated by using a conversion factor of 13:1 by weight for β‐carotene (Haskell et al., 2004; Tang, 2010). Ascorbic acid and β‐carotene analyses were performed in duplicate in 2 cans per porridge type and was only performed in Part 1 of the study.

### 2.6 Rheology

Flow properties of porridge with different formulations were determined using a hybrid rheometer (Discovery HR‐2, TA Instruments) with a parallel plate geometry (40 mm) at a gap distance 1500 µm. A small portion of porridge was loaded onto a cross‐hatched Peltier plate and subjected to a shear rate up to 100 s^−1^ (points for decade 2) at 40°C. The maximum equilibration time was set to 60 s using steady state sensing function in TRIOS software (TA Instruments, version 4.3). Curve fitting was also performed in TRIOS software, and goodness of fit was assessed based on correlation coefficient (*R*). Flow curve of all porridge samples were fitted to Carreau‐Yasuda flow model, and the viscosity at 1, 10, and 50 s^−1^ were used to estimate in‐mouth viscosity of complementary porridge for infants as described by Steele et al. (2015) and Makame et al. (2019). Each porridge sample was analysed in triplicate.

### 2.7 Sensory analysis by a trained panel

The professional sensory panel consisted of 10 subjects employed exclusively to work as sensory assessors, all women, age 44–66, not pregnant or lactating at the time of the study. The panel is highly trained, very stable, the 10 assessors are solely hired as tasters, with a part time job, and some of them have more than 30 years’ experience working with descriptive analysis. Panel performance is assessed frequently and checked for every project. That ensures that all panellists are good enough based on three important qualities: discrimination, repeatability and agreement. The panellists have been selected and trained according to recommendations in ISO 8586:2012(E). The sensory laboratory was designed according to guidelines in ISO 8589:2007(E) with separate booths, electronic registration of data (Eye Question, v. 3.8.6, Logic 8), standardised lighting and a separate ventilation system.

The porridge was received by the sensory laboratory in cans (described earlier). The References were made fresh, and flour and powder were kept at room temperature until preparation. The samples were prepared and heated 30 min before the serving, laid out in bowls and served to the assessors. The temperature of samples was 55°C ± 2°C when serving. The coded samples were tasted in blind and served following a balanced presentation design according to sample, assessor, and replicate. The samples were served in preheated porcelain bowls coded with random three‐digit codes, covered with a metal lid, and were evaluated by different modalities: odour, colour, taste/flavour and texture, more details on specific attributes in the sections below. The assessors used neutral crackers and apple boats as palate cleansers, and hot/cold water between samples.

#### 2.7.1 Quantitative descriptive analysis (QDA)

Generic QDA, based in QA, as described by Lawless and Heymann (2010), was used to describe the sensory profile of the samples. The descriptive vocabulary was created in a 1‐h pre‐trial session using two samples that stretched the sensory space under investigation. After the pre‐trial session, the attributes, definitions, and reference samples were agreed upon by the assessors. By the end of pre‐trial, all assessors were able to discriminate among samples, exhibited repeatability during trials, and reached agreement with other members of the group. For the formal assessment in the QDA, the assessors evaluated the samples in duplicate, in individual booths, during six sessions with at least 15 min break between each session. The assessors rated all attributes on non‐structured continuous scales with the left side of the scale corresponding to the lowest intensity and the right side corresponding to the highest intensity. Twenty‐four sensory attributes were evaluated by the sensory panel, including odour, taste, flavour, and texture (Appendix S1; Table S3).

#### 2.7.2 Temporal dominance of sensation (TDS)

TDS was utilised to obtain the dynamic (temporal) sensory description of the porridge samples as per Pineau et al. (2003). TDS is a multi‐attribute method in which assessors are presented with a list of sensory attributes and are asked to select the attribute perceived as dominant at each moment of the evaluation (Pineau et al., 2009). The evaluation was conducted following the approach presented in Agudelo et al. (2015). The assessors were firstly reminded the concept of dominant sensation at a given time during the food consumption, then tasted four samples (Ref 2 vs. #1, and #3 vs. #5) and listed all the attributes they perceived that were relevant to discriminate the samples in terms of dynamic perception. After that, 10 attributes were selected upon discussion and agreement among the panellists (Appendix S1; Table S4).

For the TDS the panellists evaluated the porridges in triplicate, during six sessions with at least 10 min break between each session. Assessors were instructed to put the sample in their mouth and press “START”, subsequently selecting the dominant sensations during evaluation by choosing at all times one among the 10 attributes presented in a circle on the computer screen. When the product was ready to swallow, they pressed “STOP” and spat out the sample. The assessors could successively select as many attributes as they wanted during the tasting of the samples, including re‐selecting an attribute more than once. At all times, only one attribute was selected (the dominant one).

### 2.8 Techno‐economic assessment (TEA)

A TEA was carried out to evaluate the economic performance of the process and products resulting from this study. A model was developed to estimate capital cost, operating cost, and revenue based on technical and financial input parameters. Price comparison was made with similar infant meal products in the market (Cerelac, HiPP, Bellamy and Nestlé 100–270 g).

### 2.9 Statistical analysis

The two‐tail, type 2, unpaired Student's *t*‐test in Microsoft Excel was used to determine statistically significant differences between means. This test was chosen because it is appropriate for small datasets which follows a normal distribution and have unknown variances, as in the present study. Correlation analysis was performed by the Pearson correlation method in the SigmaPlot 14.5 software. The confidence level of all analyses was set at 95%. The QDA sensory data was analysed using Principal component analysis, analysis of variance with Tukey post hoc test, and the TDS data was time standardised to remove assessor noise. The interpretation of the curves was assisted by defining a “chance level” (CL) and “significant level”, for details on these calculations see Nguyen (2019).

### 2.10 Ethical statement

The present study did not include experiments with humans or animals. No personal information, patient data or case studies were performed, and no personal data or GDPR‐related information were collected. Thus, approval from the Ethics Committee or Institutional Review Board is not needed.

## 3 RESULTS

### 3.1 Nutrients

The nutrient composition of the different porridge types is shown in Table 4.

**Table 4:** Nutrient composition per 100 g dry weight.

	Porridge no.												Recommended (reference)
	#1	#1—PF‐CP 20%	#1—PF‐CP 33%	#1—G‐CP 50%	#1—Enriched	#2	#3	#4	#5	#6	Ref 1 (FM)	Ref 2 (maize)	
Protein (g)	17.4 ± 0.01	20.1 ± 0.1	24.3 ± 0.1	20.0 ± 0.6	14.0 ± 0.1	19.6 ± 0.1	19.7 ± 0.2	19.3 ± 0.1	19.1 ± 0.1	19.7 ± 0.0	13.5 ± 0.5	17.5 ± 0.1	6–11 (Lutter & Dewey, 2003)
Fat (g)	0.3 ± 0.0	0.3 ± 0.1	0.2 ± 0.0	0.2 ± 0.1	8.2 ± 0.1	0.3 ± 0.1	0.6 ± 0.1	0.8 ± 0.1	1.5 ± 0.1	1.1 ± 0.2	0.4 ± 0.1	0.9 ± 0.0	12.7 (Lutter & Dewey, 2003)
Fiber (g)	3.3 ± 0.0	2.4 ± 0.1	2.6 ± 0.1	1.8 ± 0.1	2.5 ± 0.3	2.7 ± 0.5	1.9 ± 0.0	4.6 ± 0.3	3.2 ± 0.3	5.0 ± 1.2	2.3 ± 0.3	1.5 ± 0.2	<5 (CODEX CAC/GL 08 (1991))
Carbohydrates (g)	79.1 ± 0.1	77.3 ± 0.1	72.9 ± 0.1	77.9 ± 0.7	75.3 ± 0.2	77.6 ± 0.4	77.9 ± 0.2	75.4 ± 0.3	76.3 ± 0.4	74.3 ± 1.0	84.0 ± 0.1	80.1 ± 0.1	60–75 (CODEX CAC/GL 08, 1991)
Energy (Kcal)	388.5 ± 0.2	391.9 ± 0.7	390.6 ± 0.3	393.8 ± 0.1	431.2 ± 1.7	390.7 ± 2.1	395.2 ± 0.3	385.6 ± 1.3	394.5 ± 0.9	385.3 ± 5.5	392.8 ± 0.8	398.5 ± 0.8	440 (Lutter & Dewey, 2003)
Phosphorus (mg)	410 ± 0	458 ± 1	499 ± 3	420 ± 10	320 ± 0	520 ± 0	520 ± 0	500 ± 0	550 ± 0	555 ± 5	450 ± 10	530 ± 10	150–200 (Lutter & Dewey, 2003)
Iron (mg)	5.5 ± 0.2	8.1 ± 0.2	10.2 ± 0.2	5.3 ± 0.5	13.3 ± 0.5	3.9 ± 0.1	3.4 ± 0.0	4.3 ± 1.1	4.5 ± 0.1	5.0 ± 0.3	6.5 ± 0.1	2.7 ± 0.2	14 (Lutter & Dewey, 2003)
Potassium (mg)	1500 ± 0	1539 ± 1	1690 ± 10	1430 ± 15	1200 ± 0	1200 ± 0	1100 ± 0	1200 ± 0	935 ± 5	960 ± 20	860 ± 30	820 ± 10	516 (CODEX CAC/GL 08, 1991)
Calcium (mg)	380 ± 0	350 ± 1	304 ± 2	262 ± 8	280 ± 0	525 ± 5	515 ± 5	560 ± 0	485 ± 5	525 ± 5	590 ± 20	450 ± 10	200–400 (Lutter & Dewey, 2003)
Sodium (mg)	135 ± 5	155 ± 0	127 ± 1	98 ± 1	110 ± 0	175 ± 5	170 ± 0	150 ± 0	140 ± 0	145 ± 5	115 ± 5	130 ± 0	296 (CODEX CAC/GL 08, 1991)
Zinc (mg)	2.8 ± 0.0	3.2 ± 0.0	3.6 ± 0.0	3.1 ± 0.1	25 ± 0.0	3.2 ± 0.0	3.4 ± 0.0	3.2 ± 0.1	3.9 ± 0.1	3.8 ± 0.0	2.6 ± 0.1	3.4 ± 0.1	8.3 (Lutter & Dewey, 2003)
Vit C (mg)	15.8 ± 2.1	ND	ND	ND	ND	12.9 ± 0.2	11.8 ± 1.5	12.6 ± 0.6	10.9 ± 0.4	18.1 ± 5.6	7.8 ± 1.7	13.9 ± 1.0	140–280 (Lutter & Dewey, 2003)
β‐carotene (mg)	6.9 ± 0.1	ND	ND	ND	ND	4.4 ± 0.1	4.4 ± 1.2	2.8 ± 1.0	2.3 ± 0.8	2.3 ± 0.8	1.2 ± 0.7	1.0 ± 0.6	NA
Vitamin A (RE; µg)	530.1 ± 8.2	ND	ND	ND	ND	341.6 ± 3.9	336.9 ± 91.0	214.8 ± 78.7	177.6 ± 59.4	176.3 ± 58.1	95.6 ± 55.4	76.4 ± 45.6	500 (Lutter & Dewey, 2003)

*Note*: See Tables 2 and 3 for porridge composition.

Abbreviations: NA, not available; ND, not determined.

### 3.2 Rheology

**Figure 1:**
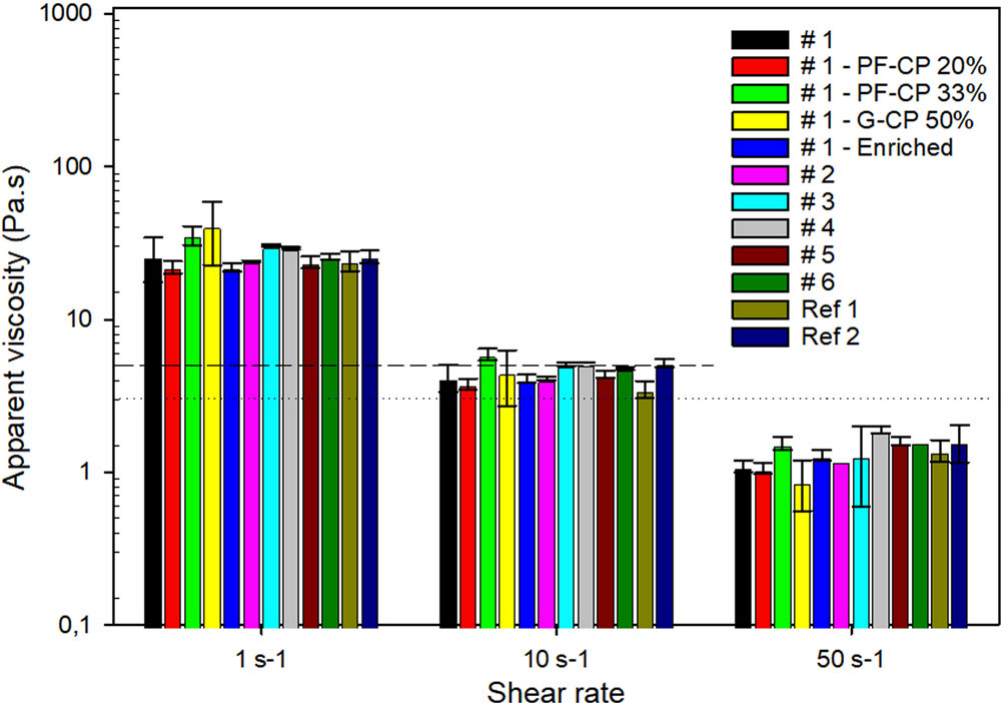
Viscosity at 3 shear rates of the complementary porridges. An oral viscosity of approx. 5 Pa.s at shear rate 10 s^−1^ and <<3 Pa.s at shear rate 50 s^−1^ when measured at 40°C is considered suitable for complementary foods. The dashed and dotted lines denotes apparent viscosity of 5 and 3 Pa.s, respectively.

The sample viscosity in this study ranged between 3.5 (Ref 1) and 5.9 Pa.s (#1—PF‐CP 33%) at 10 s^−1^ and was maintained below 3 Pa.s at 50 s^−1^ regardless of formulations, demonstrating their rheological suitability for the targeted age group of 6–23 months (Figure 1). Except for #1—PF‐CP 33% which was significantly higher than #1 at shear rate 10 s^1^ (*p* = 0.022) and 50 s^−1^ (*p* = 0.011), there were no significant difference in viscosity at any shear rate between #1 and the porridges from Part 2 (*p* ≥ 0.092). However, with 1.55 Pa.s at 50 s^−1^ #1—PF‐CP 33% was satisfactory low, i.e., <<3 Pa.s (Figure 1).

### 3.3 Sensory

**Figure 2:**
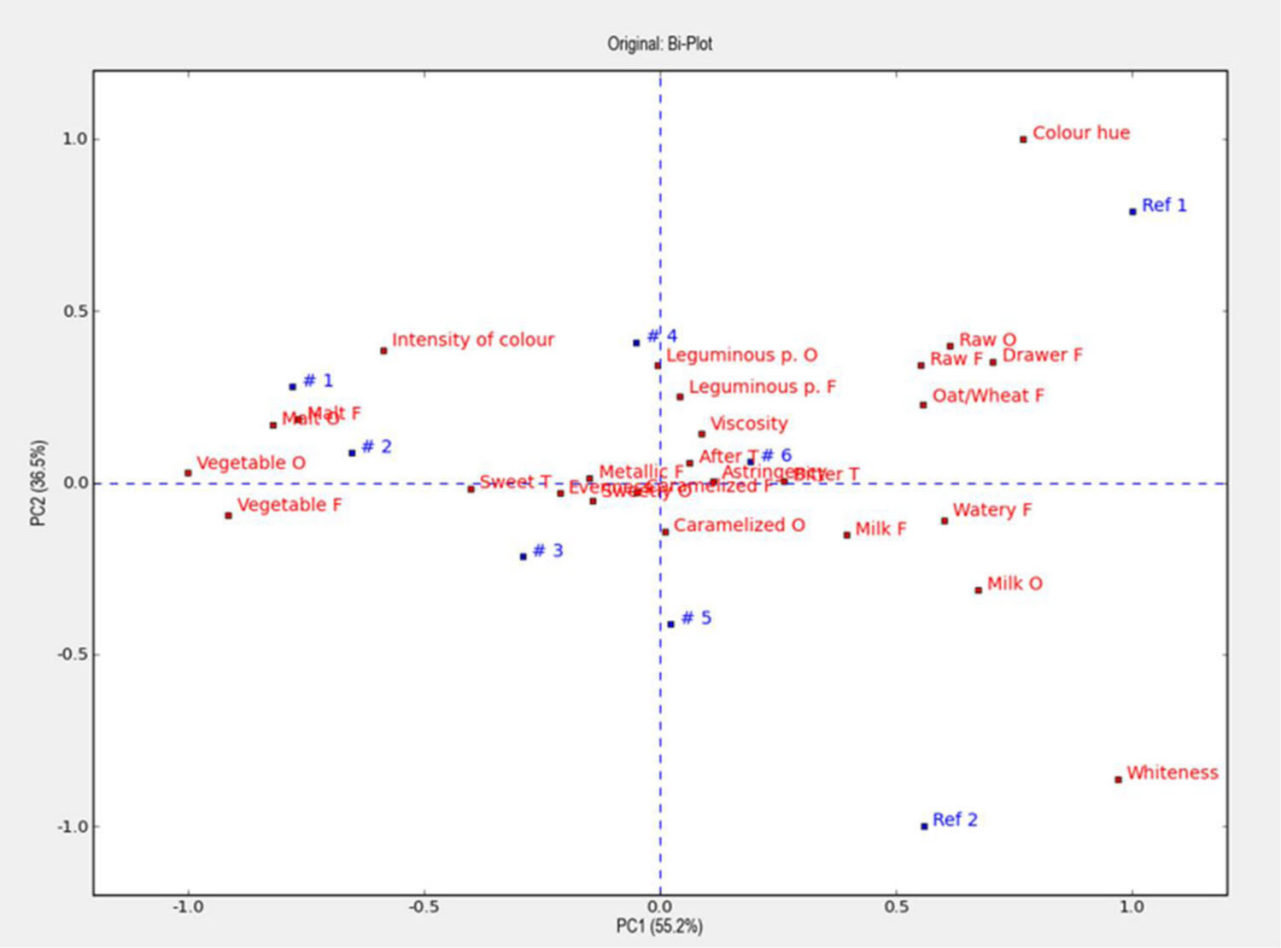
PCA—all attributes and samples. The variation of the samples can be explained to 55% of the PCA1 and to 37% of the variation is explained by the PC2. The Figure presents the odour, flavour/taste and texture scores for the eight different samples. The closer a sample is to a sensory attribute in the plot, the higher the intensity of that attribute has been measured in the respective sample. Please note that the figure should not be interpreted directly as it only illustrates the relationship between samples and attributes (O = odour, F = flavour, T = taste).

In the first trial (Part 1), the selected prototypes #1 to #6 were evaluated together with the References (Table 5 and Figure 2). The PCA plot (Figure 2) displays the sensory profiles of all samples and all significant attributes; it can be observed that the reference samples were quite distinct as compared to the prototype samples, particularly in colour, but also in vegetable, leguminous, and cereal‐related flavours. Significant differences in the individual attributes can be observed in Table 5 and are hereby summarised. Prototype #1 was perceived as significantly sweeter than both references and #4, #5 and #6, presumably as an effect of higher OFSP content, and significantly less bitter than Ref 1 and #5 and #6. Ref 1 had also significantly more raw flavour and less vegetable flavour compared to all other porridges, while #1 and #2 had significantly more vegetable flavour than both references and #6. The sensory panel was able to detect a slight but statistically significant lower leguminous flavour in Ref 2 compared to #3, #4, #6, and Ref 1. Apparently, the leguminous crops masked the milky flavour, since it was statistically significantly more pronounced in Ref 2 compared to all other porridges, and in Ref 1 compared to #1 and #2. Malt flavour correlated to the proportion of OFSP (*R* = 0.89) and was statistically significant more pronounced in #1 and #2 compared to all other porridges. Ref 2 which was made of maize was as expected perceived as significantly whiter than any other porridge and with less intense colour, whereas Ref 1 (finger millet) had significantly much higher colour hue. Perceived evenness and viscosity scored between 4.11 (#4) and 5.39 (#6), and between 4.21 (#1) and 5.39 (#3), respectively, so although significant differences were detected, these were moderate. Prototype #5 scored highest on aftertaste (6.54), which was significantly higher than the lowest scoring Ref 2 (5.51).

**Table 5:** Mean values of the sensory attributes evaluated by the trained panel in the first trial.

Attribute	Porridge no.								*p*‐value
	#1	#2	#3	#4	#5	#6	Ref 1	Ref 2	
Colour hue	3.99 BC	2.97 CD	2.79 CD	4.81 B	2.49 D	3.76 BC	7.98 A	2.05 D	<0.001
Colour intensity	4.81 A	4.44 AB	3.72 BC	3.97 ABC	3.43 C	3.57 BC	3.36 C	1.63 D	<0.001
Whiteness	2.52 D	3.24 D	5.14 BC	3.21 D	6.09 B	4.78 C	4.89 C	8.20 A	<0.001
Sweetly odour	4.59 A	4.22 A	4.02 A	3.95 A	3.87 A	3.78 A	3.76 A	4.33 A	0.054
Milk odour	1.46 E	1.64 E	1.81 DE	1.94 CDE	2.58 BCD	2.76 BC	3.09 B	4.44 A	<0.001
Raw odour	2.65 C	2.88 BC	2.74 C	3.89 BC	2.94 BC	4.04 B	5.51 A	2.91 BC	<0.001
Vegetable odour	4.72 AB	5.10 A	4.77 AB	4.14 AB	4. 02 AB	3.77 B	1.65 C	2.13 C	<0.001
Leguminous odour	3.20 A	3.01 AB	2.97 AB	3.52 A	2.94 AB	3.14 A	3.79 A	1.82 B	<0.001
Caramelised odour	1.75 A	2.01 A	2.00 A	1.71 A	2.13 A	1.81 A	1.67 A	2.37 A	0.284
Malt odour	4.01 A	3.77 A	2.36 B	2.11 BC	1.76 BC	1.69 BC	1.30 BC	1.17 C	<0.001
Sweet taste	4.81 A	4.46 AB	4.26 ABC	3.69 BC	3.71 BC	3.41 C	3.28 C	3.72 BC	<0.001
Bitter taste	5.12 B	5.31 AB	5.75 AB	5.66 AB	6.22 A	6.21 A	6.21 A	5.67 AB	0.002
Raw flavour	2.83 D	2.95 CD	3.10 BCD	4.04 BC	3.38 BCD	4.24 AB	5.38 A	3.03 CD	<0.001
Vegetable flavour	5.10 A	4.93 A	4.77 AB	4.32 ABC	4.01 ABC	3.56 BC	1.75 D	3.14 C	<0.001
Leguminous flavour	3.01 AB	3.02 AB	3.30 A	3.38 A	3.19 AB	3.23 A	3.79 A	2.10 B	0.002
Milk flavour	1.44 D	1.71 CD	2.14 BCD	1.88 BCD	2.02 BCD	2.33 BC	2.63 AB	3.19 A	<0.001
Caramelised flavour	1.57 A	1.72 A	1.66 A	1.48 A	1.60 A	1.37 A	1.46 A	1.59 A	0.836
Malt flavour	3.96 A	3.52 AB	2.48 BC	2.22 CD	1.63 CD	1.91 CD	1.37 CD	1.17 D	<0.001
Oat/wheat flavour	2.37 D	2.74 CD	3.13 BCD	3.55 BC	2.96 BCD	3.83 B	4.91 A	3.16 BCD	<0.001
Watery flavour	2.66 D	2.91 CD	3.37 CD	3.82 BC	3.88 ABC	3.67 BC	4.64 AB	4.81 A	<0.001
Drawer flavour	1.81 C	2.27 BC	2.17 BC	3.17 BC	2.44 BC	3.44 B	5.03 A	2.53 BC	<0.001
Metallic flavour	3.91 A	3.91 A	3.91 A	3.70 A	3.87 A	4.01 A	3.43 A	3.39 A	0.075
Evenness	5.27 A	4.96 A	4.49 BCD	4.11 D	5.18 AB	5.39 A	4.23 CD	4.35 CD	<0.001
Viscosity	4.21 D	5.01 AB	5.39 A	4.91 ABC	4.61 BCD	4.81 ABCD	5.40 A	4.24 CD	<0.001
Astringency	4.87 A	5.01 A	5.18 A	5.47 A	5.51 A	5.39 A	5.34 A	5.18 A	0.072
Aftertaste	5.75 AB	5.77 AB	5.87 AB	6.09 AB	6.54 A	6.34 AB	6.12 AB	5.51 B	0.018

*Note*: Different letters indicate significant differences (*p* > 0.05) according to Tukey's multiple comparison test. See Table 2 for porridge composition.

Table 6 displays the sensory profiles of the samples evaluated by the trained panel in the second trial (Part 2), as commented in the following paragraphs. Prototype #1 figured as reference for the optimised variants based on #1. The sensory panel was able to differentiate #1—G‐CP 50% as statistically significant sweeter than #1 and the two PF‐CP variants, whereas it was not significantly sweeter than #1—Enriched. #1 and #1—Enriched were statistically significant less bitter than #1—PF‐CP 33% and #1—G‐CP 50%. It may be that the modified flours contribute the bitterness through a concentration of some of the amino acids commonly found in cowpea, as e.g., phenylalanine, leucine, and tyrosine (Teka et al., 2020) which are known to produce mainly bitter flavours (Solms, 1969), but also notes of salty and metallic flavours that may also be perceived as astringent. Astringency and aftertaste gave intermediate scores all over, with values ranging 5.03–5.88, and 5.66–6.50, respectively. It should be noted, however, that #1—Enriched was significantly lower on astringency compared to the other variants except from #1. This may indicate a combination of whole milk powder rounding the astringent taste, and that the modified flours may contribute astringency as just mentioned.

**Table 6:** Mean values of the sensory attributes evaluated by the trained panel in the second trial.

Attribute	Porridge no.					*p*‐value
	#1 (reference)	#1—PF‐CP 20%	#1—PF‐CP 33%	#1—G‐CP 50%	#1—Enriched	
Colour hue	3.38 A	1.91 B	2.01 B	1.89 B	3.40 A	<0.001
Colour intensity	4.67 A	4.28 A	4.26 A	4.46 A	4.52 A	0.056
Whiteness	3.07 B	4.17 A	4.27 A	4.18 A	3.36 B	<0.001
Sweetly odour	3.69 A	4.17 A	4.16 A	4.35 A	4.24 A	0.408
Milk odour	1.86 B	2.49 AB	2.17 B	2.71 AB	3.28 A	0.004
Raw odour	4.22 A	3.71 A	4.14 A	3.31 A	3.41 A	0.179
Vegetable odour	3.73 BC	4.61 AB	4.84 A	4.01 ABC	3.23 C	<0.001
Leguminous odour	3.91 A	3.18 A	3.37 A	3.31 A	2.97 A	0.322
Caramelised odour	2.51 A	3.07 A	2.59 A	3.54 A	3.32 A	0.059
Malt odour	5.32 A	4.08 B	4.08 B	4.09 B	3.23 B	<0.001
Sweet taste	4.22 B	4.42 B	4.30 B	5.35 A	4.84 AB	0.006
Bitter taste	5.35 B	5.78 AB	6.31 A	6.53 A	5.24 B	<0.001
Raw flavour	4.37 A	3.93 A	4.12 A	3.52 A	3.69 A	0.118
Vegetable flavour	4.09 BC	5.02 A	4.79 AB	4.17 BC	3.72 C	<0.001
Leguminous flavour	3.99 A	3.32 A	3.44 A	3.86 A	3.12 A	0.280
Milk flavour	2.03 B	2.72 B	2.28 B	2.50 B	4.06 A	<0.001
Caramelised flavour	2.25 B	2.86 AB	2.64 B	3.59 A	3.67 A	<0.001
Malt flavour	5.06 A	4.19 AB	4.17 AB	4.44 AB	3.74 B	0.005
Oat/wheat flavour	2.57 A	2.53 A	2.51 A	2.99 A	3.63 A	0.125
Watery flavour	2.82 A	2.14 AB	1.94 AB	1.49 B	2.78 A	0.024
Drawer flavour	1.90 AB	1.73 AB	1.76 AB	1.38 B	3.17 A	0.053
Metallic flavour	3.33 A	3.41 A	3.21 A	2.83 A	2.96 A	0.290
Evenness	4.37 A	4.56 A	3.60 B	4.39 A	4.87 A	<0.001
Viscosity	4.89 A	4.22 A	4.72 A	4.77 A	4.03 A	0.055
Astringency	5.23 AB	5.83 A	5.87 A	5.88 A	5.03 B	0.005
Aftertaste	5.84 AB	5.75 B	5.80 AB	6.50 A	5.66 B	0.016

*Note*: Different letters indicate significant differences (*p* > 0.05) according to Tukey's multiple comparison test. See Tables 2 (#1) and 3 for porridge composition.

The two PF‐CP variants had the most vegetable flavour; #1—PF‐CP 20% was significantly higher than all except #1—PF‐CP 33%, which again was significantly higher than #1—Enriched, which had the least vegetable flavour, but not significantly less than #1 and #1—G‐CP 50%. The same trend was also observed for vegetable odour. Milk flavour and odour were as expected highest in #1—Enriched, which was the only variant containing whole milk powder. The milk flavour was significantly higher than in all other variants, and milk odour was significantly higher compared to #1 and #1—G‐CP 50%. #1, on the other hand, demonstrated the lowest scores for milk flavour and odour, however, only significantly less than #1—Enriched. #1 had the highest malt flavour and odour; malt odour was significantly higher than in any other of the variants, and malt flavour was significantly higher than in #1—Enriched, indicating that whole milk powder may mask malt flavour that is presumably contributed by OFSP. #1—Enriched, and #1—G‐CP 50% had both significantly more caramelised flavour than #1, which may be a preferred taste for infants and young children, however, there was no statistically significant difference in caramelised odour. #1 and #1—Enriched were perceived as significantly waterier than #1—G‐CP 50% but the scores for this attribute were all generally low (≤2.82). The three variants containing modified cowpea were all perceived as significantly whiter than #1 and #1—Enriched. #1—PF‐CP 33% was perceived as significantly less even than all other variants.

#### 3.3.1 Temporal dominance of sensations

**Figure 3:**
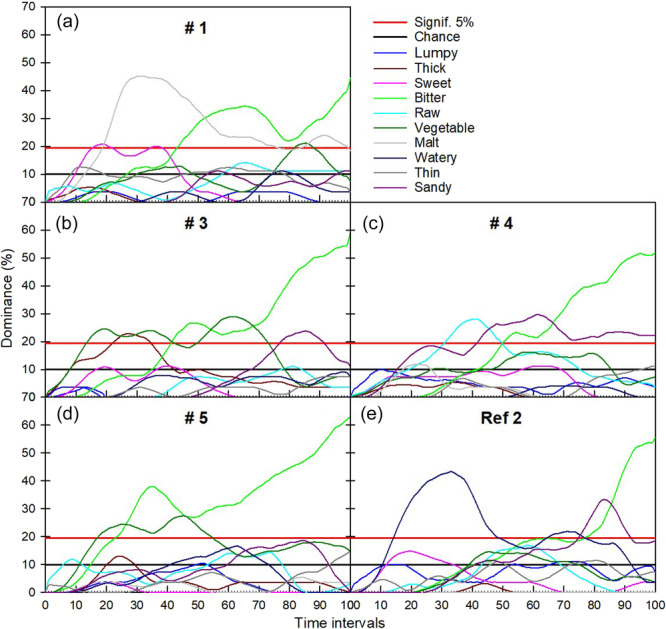
Temporal dominance of sensations (TDS) plots of prototypes #1 (a), #3 (b), #4 (c), #5 (d), and Reference #2 (Maize) (e). Curves display the dominance rate (%) versus the standardised evaluation time in seconds. The black line marks the chance level (CL) and the red line marks the significance level (LS).

Prototypes #1, #3, #4, #5, and Reference #2 (Maize) were selected for the TDS. The samples were selected based on the QDA results to represent the whole sensory space, looking into reducing the number of samples to be evaluated by the panel, TDS evaluation is demanding for panellists, and is more resource demanding than QDA, as at least three repetitions are required. The attributes generated by the panellists, were related to the main sensory characteristics of the products that allow differentiating their dynamic profiles. On the TDS plots (Figure 3), two lines were drawn for the CL and significance levels (LS). The CL refers to the dominance rate that an attribute could obtain by chance and the LS is the minimum value this proportion should equal if it is to be considered significantly (*p* < 0.05) higher than the CL (for details on the calculations of CL and LS, see Pineau et al. (2009).

TDS curves allow to understand the temporal aspects of perception (during consumption), allowing a better understanding of the dynamics of perception. Figure 3 displays the TDS curves of some of the prototypes and Ref 2. The dynamic profiles of the five samples were quite distinct, but all of them were dominantly bitter at the end of the consumption. Sample #1 was dominantly malty in the first half of its consumption, adding to the information from the QDA that described #1 as with significantly more malty in odour and flavour than the rest of the samples (together with #2 and correlated to OFSP content), this malty perception, was more apparent at the beginning of the consumption. Sample #3 had no clear dominance of one single attribute in the first part of the consumption, with many attributes just reaching the LS (vegetable, thick, lumpy, bitter), and at the end of the consumption dominantly bitter and somehow sandy. Sample #4 was dominantly raw and sandy in the beginning of the consumption, the sandiness persisted towards the end, but with a higher dominance of bitterness. Interestingly, QDA did not unveil differences in the intensity of raw flavour, but it was highlighted as dominant in #4. Sample #5 was at first dominantly vegetable, but dominantly bitter in most of the consumption period. Ref 2 was quite different in dynamic profile, dominantly watery in the first half of the consumption, and sandy and bitter in the end. Ref 2 showed a high intensity of water in the QDA results, with the TDS showing that this sensation appears dominantly at the beginning.

That all samples were dominantly bitter at the end of the consumption is in agreement to the QDA profiles, showing that all samples were similarly intense in terms of bitterness and astringency, but the TDS results suggesting that this sensation was more apparent at the end of the consumption, in agreement with the QDA rating of intense aftertaste. Sandiness was not selected by the panel as an important descriptor for the static sensory profiles (via QDA) but was selected as relevant to describe the temporal perception, with samples #3, #4 and Ref 2 dominantly sandy at the end of the consumption; this can be related to the addition of Bambara groundnut in #3 and #4 and the significantly higher content of maize in Ref 2.

### 3.4 Techno‐economics assessment (TEA)

A detailed TEA of the start‐up business was carried with a 10‐year forecast. The discounted cash flow as shown in Appendix S2 indicates that the business will break even in the third year of operation with an Internal Rate of Return of 42% and a Net Present Value (NPV) of Euro 2,214,963. This clearly indicates that the business is a profitable business.

## 4 DISCUSSION

### 4.1 The choice of canning for complementary food processing

Canning provides a long shelf life without using preservatives. The metal can withstand the high thermal load applied, and it is very robust and not prone to breakage. The thermal load applied in the present study will kill all vegetative bacteria as well as spores of pathogenic bacteria that may be present in the raw material, including the very heat resistant spores of *Clostridium botulinum* (Jay, 1998). The metal cans may be substituted with e.g. cardboard cartons or glass jars, and the principles of preserving the food will remain. However, glass and cardboard may allow light to penetrate to a greater extent than metal cans. Light‐induced changes includes e.g. the degradation of vitamins A and C and photosensitised oxidation (Bekbölet, 1990). The circular form of the can enables more uniform heat distribution than the cardboard carton, in which burning may be a problem. Glass jars need slower heating and cooling rates to avoid breakage, and it is also necessary to ensure a vacuum, which can be achieved by hot filling, vacuum filling or steam flushing. These are steps that may negatively affect time and cost efficiency compared to canning in metal cans. Alternatively, the flour formulations may also be packaged as dry flour and left for the consumers to reconstitute before feeding. However, this strategy does not ensure as efficient inactivation of potential pathogens in the raw material without adding complicated and costly decontamination measures, and there may also be a scarcity of safe potable water in some regions. Combined with the process technology proposed in the present study, the metal cans allow for a very long shelf life (years) at ambient temperatures and is superior in terms of food safety and convenience. Thus, the product envisaged may be considered for emergency stocks.

### 4.2 Nutrients

The protein content in all porridges was satisfactory and well above recommended limits (Table 4). However, protein was increased by 2.7%–6.9% by using modified cowpea flours, leading to statistically significant higher protein content in #1—PF‐CP 20%, #1—PF‐CP 33%, and #1—G‐CP 50%, compared to #1 (*p* ≤ 0.021). The amino acids profiles were not determined. However, it may be assumed that porridges containing a mix of legumes and cereals can result in a balanced amino acid profile as they have different limiting amino acids. The energy density in the proposed porridges is below the recommended 440 kcal/100 g dw (Lutter & Dewey, 2003). Except for #1—Enriched, the energy content is approx. 390 kcal/100 g dw which is also close to what is found in both reference porridges made from maize and finger millet. The enriched porridge (#1—Enriched; Table 4) is statistically significant (*p* ≤ 0.003) higher than all other porridges including both references and approaching the recommended level (440 Kcal/g dw) with a value of 431.2 Kcal per 100 g dw. From the age of 6 months, complementary feeding becomes necessary because the infants need for energy and nutrients starts to exceed what can be provided by breast milk alone (Dewey & Brown, 2003). The energy needed in addition to breast milk is about 200, 300, and 550 Kcal per day, in infants 6–8 months, 9–11 months and 12–23 months of age, respectively (World Health Organization, 2000). Complementary foods should have a greater energy density than breast milk, and at least 0.8 Kcal per gram (World Health Organization, 2009), and with this level, it is recommended that the feeding frequency should be 2–3 meals per day at 6–8 months, and 3–4 at 9–24 months of age, and to include additional nutritious snacks once or twice per day, depending on the child's hunger and satiety (Dewey & Adu‐Afarwuah, 2008). Considering this, children in the younger age group need to consume approx. 380 g (#1—Enriched) to 420 g wet porridge per day to meet the recommended daily energy levels. This may sound a lot, but considering an appealing consistency and taste, it may be possible to achieve when divided on 3 meals. The average recommended amount of complementary food serving for a child aged 6–24 months is 50 g per meal (Lutter & Dewey, 2003). However, the amount is higher for cereal and plant based foods compared to meat, and also higher for low viscosity foods with high water content (CODEX CAC/GL 08, 1991). Recent studies have shown that amounts of well over 50 g and up to ~150 g were consumed by children in the age group per meal when the complementary food was well accepted (Atsbha, 2021; Bankole et al., 2023; Buzigi et al., 2020). The average complementary feeding frequency for the age group 6–9 months in developing countries is 3–3.6 times per day, and more frequently as the child grows older (Dewey et al., 2006). However, continued breastfeeding and supplementation by preferably animal source foods is strongly advised and will reduce the volume of porridge needed. Several studies strongly advise to regularly provide an animal‐source food to the infant (Dewey & Adu‐Afarwuah, 2008; Guldan et al., 2000; Penny et al., 2005), which will also enhance micronutrient intake of e.g. iron and zinc. It was reported that Bambara groundnut composite porridges were enriched in protein and energy, and had a lower viscosity compared to maize porridges (Kikafunda et al., 1997; Temba et al., 2017). Indeed, the two proposed porridges, #3 and #4, which both contained 11.5% Bambara groundnut on a dry matter basis, were slightly and statistically significant (*p* ≤ 0.006) higher in protein compared to the maize reference, but no evident improvement was observed in neither energy content (*p* ≥ 0.058) nor viscosity (*p* ≥ 0.261).

The recommended carbohydrate level in complementary foods is 60–75 g per 100 g dw (CODEX CAC/GL 08, 1991), and all proposed prototypes are slightly above this level. Carbohydrates may be provided from several sources and differ greatly in their content of nutrients. Preferably, carbohydrates should not be in the form of added sugar, but be complex and sourced from legumes, vegetables, grains, and nuts (Lutter et al., 2021), as in the present proposed prototypes.

Breast milk is usually rich in fat, so little additional fat from complementary foods is needed while breast milk intake is still high. However, the fat content gets more important as breast milk intake declines with the child's age (Dewey & Adu‐Afarwuah, 2008). The fat content of the proposed porridges except for #1—Enriched, is far below the recommended limit, again highlighting the need for an animal‐source food. Based on calculation, we found that fat content could be significantly increased by using whole milk powder instead of skimmed milk powder, however, it would still be below the recommended levels. Inclusion of a vegetable oil like e.g., sunflower oil would further increase fat content significantly. As shown by Amagloh and Coad (2014) addition of full‐fat soybean flour, soybean oil, and anchovy powder to a complementary food consisting of ~90% OFSP resulted in satisfactory levels of fat, energy, protein, vitamin A, and several other micronutrients. Indeed, the modification of #1 resulting in #1—Enriched, did in fact increase fat more than 20‐fold, to 8.2 g per 100 g dw, resulting in 431.2 Kcal per 100 g dw, approaching the proposed limits of 12.7 g fat and 440 Kcal per 100 g dw, respectively.

Because the dietary fibres are slowly absorbed and fermented by intestinal flora, thus causing a laxative effect, the crude fibre content of the complementary food should not exceed 5 g per 100 g dry matter (CODEX CAC/GL 08, 1991), and the intake per day should be 10 g/day for children 1–3 years (EFSA Panel on Dietetic Products Nutrition and Allergies NDA, 2010). Accordingly, the fibre content in the proposed porridges is satisfactory (Table 4). A complementary feeding diet with a high content of crude fibre may decrease energy density (Lutter et al., 2021).

Iron and zinc were both far below the recommended limits, except for in the supplemented #1—Enriched (Table 4). When zinc deficiency is reported, it is often followed by nutritional iron deficiency because iron and zinc have a similar distribution in the food supply, and their bioavailability is regulated in a similar way (Gibson & Ferguson, 1999). Indigenous Ethiopian teff is reported to be very high in iron as a result of contamination from soil as a result of traditional threshing methods (Abebe et al., 2007). Thus, nutritional iron deficiency is not a serious problem in Ethiopia due to the consumption of the indigenous high‐iron cereal teff and some oleaginous seeds and kale, all relatively high in iron (Abebe et al., 2007). The light teff flour used in this study was not of Ethiopian origin, and low in iron (5.7 mg/100 g according to declaration) compared to what is normally found in Ethiopian indigenous teff (Baye, 2014). Consequently, #1, #2, and #3, which contained the most teff, did not stand out compared to the other prototypes in terms of iron or zinc. The iron content in these 3 prototypes were statistically significantly higher than that in the maize reference (*p* ≤ 0.036), but significantly lower than in the finger millet reference (*p* ≤ 0.028). Iron and zinc have been previously shown to be enriched in the protein‐rich fraction of pulses during air‐classification (De Angelis et al., 2021). Indeed, #1—PF‐CP 20% was statistically significant higher in iron (*p* = 0.002) and zinc (*p* < 0.0001) compared to its counterpart #1 and increasing the PF‐CP content further from 20% to 33% led to further statistically significant increases in both iron (*p* = 0.0005) and zinc (*p* < 0.0001), however, still relatively far below recommended levels (Table 4).

Iron, zinc, and calcium are limiting nutrients in unfortified plant‐based complementary foods commonly used in developing countries (Abeshu et al., 2016; Dewey & Brown, 2003; Gibson et al., 1998; World Health Organization, 2009). According to Baye et al. (2013), calcium in the complementary foods collected from the Amhara region, Ethiopia, was below the desired values, but iron and zinc were within the recommended limits when accounting for low or moderate bioavailability. While the children consumed enough protein and iron, they fell short of their calcium and zinc needs from complementary foods. In contrast to this, Gibson et al. (1998) reported that the complementary foods under study failed to meet the desirable level for calcium, zinc, and iron, even if moderate bioavailability was assumed for iron and zinc, and would not satisfy the estimated daily requirement for these nutrients for children of 9–11 months, even if ideal serving sizes were provided. Ryckman et al. (2021) identified complementary feeding gaps in iron and vitamin A in all of six countries under study in Eastern and South Africa, and zinc gaps in Ethiopia and Zambia. According to Dewey and Brown (2003), the amounts of iron and zinc in complementary foods are often too low without fortification, and strategies to alleviate this shortcoming may include stimulating to more breastfeeding, provide an animal‐source food, micronutrient fortification, or fortification with e.g. ascorbic acid to enhance bioavailability. Zinc is an economically limiting nutrient, and it is difficult to improve zinc intake by dietary changes without significantly increasing the cost of the food. Seafood and meat, which are rich in zinc, are often too expensive or unavailable, and would thus not solve the problem. Indeed, it was shown that a low‐cost porridge for children could be obtained by including supplement of zinc, and that zinc supplement actually reduced the cost significantly compared to when zinc was obtained through other sources (De Carvalho et al., 2015). By supplementing with zinc and iron according to De Carvalho et al. (2015), we achieved more than twofold increase in iron and ninefold of zinc (Table 4; #1—Enriched), approaching the proposed level for iron and over‐shooting the proposed level for zinc. However, there are indications that complementary foods fortified with multiple micronutrients, including zinc, have little impact on plasma zinc concentration, perhaps because of the relatively low bioavailability of zinc when consumed with cereal‐based foods (Dewey & Adu‐Afarwuah, 2008).

Phosphorus, potassium, and calcium were all well over the recommended limits in all porridges including the references, whereas sodium was below (Table 4).

β‐carotene is a fat‐soluble plant pigment found in red, orange, and yellow vegetables and fruits. β‐carotene is converted to vitamin A when the body is in short supply and is the main source of vitamin A for most people in the world (Burri, 1997; Johnson & Russell, 2004). The occurrence of vitamin A deficiency among infants in sub‐Saharan Africa is a public health concern (Amagloh & Coad, 2014; Burri, 1997). There is no published recommended limit for β‐carotene in complementary foods, however, the limit for vitamin A, as REs is 500 µg per 100 g dw (Lutter & Dewey, 2003). The content of β‐carotene correlated well to the proportion of OFSP with a correlation coefficient (*R*) of 0.824 (*p* < 0.005). However, there was no correlation between vitamin C and OFSP (*R* = 0.281, *p* = 0.119). Notably, the formulations with the highest proportions of OFSP (#1, #2, and #3) were significantly higher in β‐carotene than both references (*p* ≤ 0.009), and in #1, the estimated RE amount is over the recommended level and more than fivefold higher than in the references (Table 4). These results are in line with those of Amagloh and Coad (2014) in that OFSP‐based complementary foods is a good source of β‐carotene and may improve the vitamin A status of infants more than e.g., maize‐based foods. This implies that it should be easy to fulfil vitamin A requirement by including OFSP as a main ingredient in porridge recipes, and vitamin A deficiency will then not be caused by economic constraints if OFSP becomes more acceptable. Thus, it may be argued that supplementation is necessary for zinc and possibly iron, but not for vitamin A (De Carvalho et al., 2015; Ryckman et al., 2022). OFSP‐based porridge also has lower viscosity and aflatoxin level compared to cereal‐based complementary blends (Amagloh, 2022).

Prototype #1—Enriched was undoubtedly the best‐performing variant regarding nutrient content (Table 4). With the presumption that it contains comparable vitamin C and β‐carotene levels as #1 which it is based on, it fulfils, or approach recommended levels on all parameters analysed except for sodium and vitamin C. Since neither sodium salts nor ascorbic acid are considered cost‐limiting nutrients and are readily available, it may be argued that such fortification may be performed without significantly increasing the price.

### 4.3 Rheology

Regarding the determination of complementary porridge flow properties, there are currently no clear scientific specifications, but there is an agreement that viscosity of approx. 3 Pa.s or less within the 10–100 s^−1^ range is recommended to ensure oral processability in the age group (Makame et al., 2020; Treche, 1999; Trèche, 2001). More specifically, a viscosity of <<3 Pa.s has been recommended at shear rate 83.2 s^−1^, which is often the standard when using the Haake VT500 viscometer, for children in the age group 9–11 months (Treche, 1999), or less than 3 Pa.s at 40°C and shear rate 50 s^−1^ (Makame et al., 2020; Rombo et al., 2001; Thaoge et al., 2003) for children under the age of 3 years. It has been recommended to analyse flow properties at several and lower shear‐rates to better evaluate the oral processing abilities of food intended for infants and people with dysphagia (Makame et al., 2020; Steele et al., 2015). Germination has been identified as one of the means to reduce viscosity of complementary foods, and this is as an effect of enzymatic activity because alpha‐ and beta‐amylases are activated upon germination (Nnam, 2001). This effect is evidenced by the relatively low viscosity of #1—G‐CP 50% with a solids content of 18.2% compared to 12.2% for #1 (Figure 1).

### 4.4 Sensory

Regarding the sensory profiles, the reference samples were quite distinct as compared to the prototype samples. Wider differences were described by the panel mostly in colour, while moderated differences were highlighted in vegetable, leguminous, and cereal‐related flavours as well as textural attributes as evenness and viscosity. Attributes generally perceived as negative, like astringency or metallic, were non significantly different among references and prototypes or significantly less intense in the prototypes, for example in drawer and watery flavours. The obtained results are encouraging, as previous studies found that familiarity, texture, and colour were the most significant drivers of liking in processed sorghum and sweet potato porridges (Nnam, 2001), with familiarity with traditional food properties playing a crucial role in shaping the preferences. They also highlight the importance of viscosity as a driver of acceptance, and the prototypes in the study were perceived as with similar viscosities to the references. Our results found that the prototypes imparted some positively perceived attributes like sweet taste, malty odour and flavour and evenness. In these lines, Saka et al. (2018) found that maize porridges achieving the right balance of texture (creaminess, smoothness) and sweetness, could significantly improve the sensory appeal of maize–soy porridges for both mothers and young children. Other previous studies highlight positive aspects of the ingredients used in our study, in line with our results, e.g. OFSP flour adding natural sweetness and a vibrant colour that appeals to both children and adults, enhancing the porridge's overall acceptability (Tumuhimbise et al., 2019). Cowpea flour contributes a creamy texture, well‐regarded for improving mouthfeel and aroma (Makame et al., 2020); prototype #1 and #2 with cowpea were found as highly even and #2 was one of the most viscous samples. Bambara groundnut flour provides a rich, earthy flavour and a thicker consistency, which are appreciated for their complexity and satisfying nature (Kobue‐Lekalake et al., 2022), and were also rated among the most viscous in our study. Lastly, finger millet flour may impart smooth texture that increases consumer appeal (AA et al., 2021), evenness was significantly higher in prototype #6. Overall, the described sensory aspects, suggest the prototype porridges would be highly acceptable and nutritious options for various demographic groups.

### 4.5 Techno‐economics assessment (TEA)

A TEA of a hypothetical African company was carried out for two purposes; to assess the profitability for industrial production of the proposed canned complementary porridges, and to assess the customer's affordability of the resulting industrially processed products. These two factors are of course closely linked, and the price that the customer will have to pay depends on raw material prices, the price of equipment and facilities, the level of wages for workers, and the level of profit that the company will make. Products in the market with traditional complementary foods (Nnam, 2001) have fewer nutritional components and the prices of all the infant porridge formulations of the present study were significantly cheaper than those of the competitors in the market. This is very encouraging because the infant formulation could be affordable to families in Africa. Supplementation as proposed for #1—Enriched results in minimal (mg) addition into the formulation. Even if a price of 10 Euro per kg is assumed for zinc citrate and iron sulphate added as supplements, the price increase poised by the proposed supplementation will have little negative effect on consumers private economy but may have positive effects on the health and wellbeing of infants, and thus a positive societal economic impact. For East African families, food expenditure account for up to 65% of total expenditure and affordability likely presents substantial barriers to fulfilling most nutrient gaps among poorer households (Ryckman et al., 2021). Maize flour is a cheap staple and is therefore frequently used as main or sole ingredient in complementary food although its nutritional quality may not be satisfactory. Although for example OFSP may be comparably expensive, it is an affordable source of vitamin A, because relatively small amounts are required to satisfy the need. It is therefore possible to utilise blends of flours from affordable raw materials and supplement with few limiting nutrients, like iron and zinc, to industrially produce nutritious and affordable complementary foods.

## 5 CONCLUSIONS

Nutritious complementary foods are expensive, especially those which include animal proteins and are sufficiently rich in essential minerals and vitamins. Thus, complementary foods in Africa are often plant based, contributing to an excess frequency of e.g., zinc, iron, and vitamin A deficiency in infants and children. The present study shows a potential for industrial production of canned complementary foods based on indigenous African plant raw materials. However, there is a need to supplement with fat‐rich ingredients like whole milk powder and/or vegetable oils to ensure sufficient energy, and to further fortify the cost‐limiting nutrients zinc and iron since it is seemingly difficult to improve dietary intake of these mineral nutrients without significantly increasing the cost of the food. The prototype presented here which is based on OFSP, cowpea, and teff, enriched in iron and zinc, and amended with whole milk powder and sunflower oil (#1—Enriched), approach or fulfils recommended levels for the ‘problem nutrients’ zinc, iron, and vitamin A, and protein, energy, and other nutrients analysed (fat, fibre, phosphorus, potassium, calcium), except for sodium and vitamin C. We propose that the prototype can be further enriched in the lacking nutrients without significantly increasing the price, considering that sodium and ascorbic acid are not cost‐limiting nutrients. As add‐ons, the prototype is also gluten free and without added sugar. Our TEA suggests that it is possible to industrially manufacture the product at a retail price that is appealing to households that are income constrained. The prototypes also fall within the recommended range regarding consistency for infants as young as 6 months. Affordable, African indigenous/local complementary porridges normally do not meet the same low viscosity when prepared to meet the reference nutrient intakes, and vice versa, thus contributing to child malnutrition. From a sensory perspective, the enriched prototypes were not significantly different in consistency from the traditional porridges, with comparable levels of bitterness, metallic and astringency (usually perceived as negative attributes), differing in colour and flavour and imparting some usually positively perceived attributes like sweet taste, malty odour and flavour and evenness.

## AUTHOR CONTRIBUTIONS

Trond Løvdal, Paula Varela and Josefine Skaret contributed to the conception and design of the work. Trond Løvdal, Gorana Drobac, Blessed Okole, Josefine Skaret, and Natalia Rosa‐Sibakov performed the experimental work. Natalia Rosa‐Sibakov, Trond Løvdal and Paula Varela contributed to funding acquisition. All authors contributed to the analysis and interpretation of the data. Trond Løvdal, Paula Varela, Josefine Skaret, Izumi Sone, Natalia Rosa‐Sibakov and Blessed Okole drafted the paper, and all authors contributed with writing and editing. All authors approved the final version of the paper.

## CONFLICT OF INTEREST STATEMENT

The authors declare no conflicts of interest.

## Supporting information

Supporting information.

Supporting information.

## Data Availability

The data that support the findings of this study are available from the corresponding author upon reasonable request.
