# Supplementary material for: Canned complementary porridges for infants and young children (6–23 months) based on African indigenous crops; nutritional content, consistency, sensory, and affordability compared to traditional porridges based on maize and finger millet
Source: Matern Child Nutr. 2024 Nov 5;21(1):e13752. doi: 10.1111/mcn.13752 (PMC11650055; doi:10.1111/mcn.13752)
Supplement: Supplementary file 1 — Supporting information. [file MCN-21-e13752-s001.docx]

**Supplementary Appendix 1**

*Supplementary Table 1. Macro nutrients of flour types used for complementary porridge prototypes.* *OFSP=Orange Fleshed Sweet Potato, CP=Cowpea, BGN=Bambara Groundnut, FM=Finger Millet.*

| Flour type | Crude protein (%) | Crude fibre (%) | Crude fat (%) | Carbohydrates (%) | Energy * (Kcal/100 g) |
| --- | --- | --- | --- | --- | --- |
| OFSP | *8.1* ^Δ^ | 2.6 (Rodrigues, Barbosa Junior, & Barbosa, 2016) | *0.6* | *88.7* | 392.6 |
| CP | *21.1* | *6.6* | *0.9* | *71.4* | *378.1* |
| BGN | *17.7* | *5.2* | *6.0* | *71.1* | *409.2* |
| Teff † | 12.3 | 7.9 | 2.0 | 70.0 | 367.0 |
| Maize | *8.5* | *5.7* | *4.7* | *81.1* | *400.7* |
| FM | *10.1* | *18.7* | *1.2* | *70.0* | *331.2* |
| Amaranth | *17.0* | *7.1* | *2.9* | *73.0* | *386.1* |

^Δ^ Values in italics denotes data analysed in this study.

† Values as declared by the provider

* Calculated Energy (Kcal/100 g sample) protein × 4 + fat × 9 + carbohydrates × 4

*Supplementary Table 2. Macro nutrients of germinated cowpea (G-CP) flour and the protein-rich fraction of cowpea (PF-CP) flour.*

| Flour type | Crude protein (%) | Crude fibre (%) | Crude fat (%) | Carbohydrates (%) | Energy * (Kcal/100 g) |
| --- | --- | --- | --- | --- | --- |
| G-CP | *26.2* | *1.2* | *1.5* | *71.1* | *402.7* |
| PF-CP | *43.5* | *1.2* | *4.6* | *50.7* | *418.2* |

* Calculated Energy (Kcal/100 g sample) protein × 4 + fat × 9 + carbohydrates × 4

Definition of sensory attributes

Sensory attributes for profiling of quantitative descriptive analysis (QDA) and temporal dominance of sensations (TDS) are presented in Supplementary Tables 3 and 4, respectively.

*Supplementary Table 3. Definition of sensory attributes used in sensory profiling – QDA.*

|  | Attribute | Description |
| --- | --- | --- |
| Colour | Colour hue | Colour assessed on whole berries according to the Natural Colour System (NCS); No intensity = Y20R (yellow), high intensity = Y20B (red/blue) |
|  | Colour intensity | Colour intensity on the surface according to NCS |
|  | Whiteness | Colour assessed on the surface according to NCS |
| Odour | Sweetly odour | Relates to all sweet odours |
|  | Milk odour |  |
|  | Raw odour | A raw odour related to undercooked / flour etc. grain |
|  | Vegetable odour | Related to a smell of root vegetables (such as Jerusalem artichoke, potato, sweet potato, carrot) |
|  | Leguminous odour | Related to an odour reminiscent of legumes (peas, beans, lentils) |
|  | Caramelized odour | Related to an odour of caramelization |
|  | Malt odour | Related to the odour of malt |
| Flavour/Taste | Sweet taste | Related to the basic taste sweet (sucrose) |
|  | Bitter taste | Related to all bitter tastes |
|  | Raw flavour | A raw taste related to lightly cooked / flour, grain etc. bran |
|  | Vegetable flavour | Related to a taste of root vegetables (courgette, potato, sweet potato, carrot) |
|  | Leguminous flavour | Related to a taste reminiscent of legumes (peas, beans, lentils) |
|  | Caramelized flavour | Related to a flavour of caramelization |
|  | Malt flavour | Related to the flavour of malt |
|  | Oat/wheat flavour | Related to the taste of oats and wheat |
|  | Watery flavour | Related to a watery, watered-down flavour |
|  | Drawer flavour | Related to the taste of drawer |
|  | Metallic flavour | Related to the flavour of metallic (ferrous sulfate) |
| Texture | Evenness | Geometric texture attribute related to the sensory perception of particle size and particle shape in a product |
|  | Viscosity | Mechanical texture attribute relating to resistance to flow. It corresponds to the force required to draw a liquid from a spoon over the tongue |
|  | Astringency |  |
|  | Aftertaste |  |

*Supplementary Table 4. Definition of sensory attributes used in TDS.*

|  | Attribute | Description |
| --- | --- | --- |
| Flavour/Taste | Sweet taste | Related to the basic taste sweet (sucrose) |
|  | Bitter taste | Related to all bitter tastes |
|  | Raw flavour | A raw taste related to lightly cooked / flour, grain etc. bran |
|  | Vegetable flavour | Related to a taste of root vegetables (courgette, potato, sweet potato, carrot) |
|  | Malt flavour | Related to the flavour of malt |
|  | Watery flavour | Related to a watery, watered-down flavour |
| Texture | Thin | Mechanical textural attribute relating to resistance to flow. It corresponds to the force required to draw a liquid from a spoon over the tongue |
|  | Thick | Mechanical textural attribute relating to resistance to flow. It corresponds to the force required to draw a liquid from a spoon over the tongue |
|  | Lumpy | A lumpy sensation in the mouth |
|  | Sandy | A sandy sensation in the mouth |

References

Rodrigues, N., Barbosa Junior, J. L., & Barbosa, M. I. M. J. (2016). Determination of physico-chemical composition, nutritional facts and technological quality of organic orange and purple-fleshed sweet potatoes and its flours. *International Food Research Journal, 23*(5), 2071-2078.
